# Epidemiology of Malaria in Endemic Areas

**DOI:** 10.4084/MJHID.2012.060

**Published:** 2012-10-04

**Authors:** Beatrice Autino, Alice Noris, Rosario Russo, Francesco Castelli

**Affiliations:** 1 University Division of Infectious and Tropical Diseases, University of Brescia and Spedali Civili General Hospital, Brescia (Italy), Piazza Spedali Civili, 1 - 25123–Brescia, Italy; 2 University Division of Infectious Diseases, University of Catania, Via Palermo 635 – 95100 Catania, Italy; 3 Chair of Infectious Diseases, University of Brescia, Italy

## Abstract

Malaria infection is still to be considered a major public health problem in those 106 countries where the risk of contracting the infection with one or more of the Plasmodium species exists. According to estimates from the World Health Organization, over 200 million cases and about 655.000 deaths have occurred in 2010. Estimating the real health and social burden of the disease is a difficult task, because many of the malaria endemic countries have limited diagnostic resources, especially in rural settings where conditions with similar clinical picture may coexist in the same geographical areas. Moreover, asymptomatic parasitaemia may occur in high transmission areas after childhood, when anti-malaria semi-immunity occurs. Malaria endemicity and control activities are very complex issues, that are influenced by factors related to the host, to the parasite, to the vector, to the environment and to the health system capacity to fully implement available anti-malaria weapons such as rapid diagnostic tests, artemisinin-based combination treatment, impregnated bed-nets and insecticide residual spraying while waiting for an effective vaccine to be made available.

## Introduction

Malaria is one of the most important public health problem in term of morbidity and mortality, causing more than 200 million cases and 655.000 deaths every year.^1^

**Figure 1:**
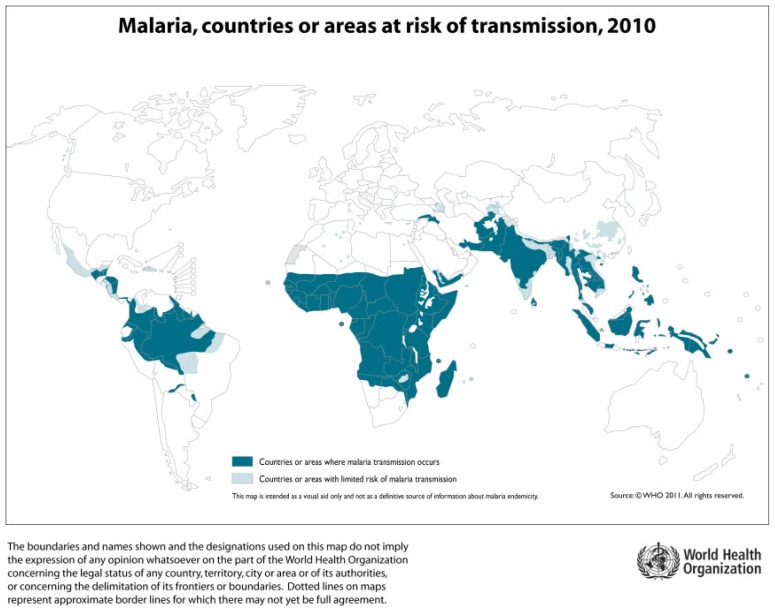
Distribution of territories at risk of malaria transmission, 2010 (www.gamapserver.who.itn/maplibrary/Files/Map/Global_Malaria_2010.png)

According to the World Health Organization (WHO) Malaria Report 2011, a total of 106 countries in the world are at risk of transmission of malaria infection (Figure 1).

A total of 216 million estimated malaria cases occurred in 2010, 81% of which were reported in the African Region, followed by South East Asia (13%) and Eastern Mediterranean Region (5%). The total number of malaria deaths was estimated to be 655.000 in 2010; 91% of whom occurred in the African Region, 6% in South-East Asia and 3% in Eastern Mediterranean Region (Table 1).

Although the proportion of people exposed to malaria parasites has decreased during the last century, the absolute number of people at risk for malaria infection increased from 0.8 billion in 1900 to 3.3 billion in 2010, as a consequence of the absolute increase of the population living in malaria-endemic regions.^1^,^2^ However, between 2005 and 2010 malaria cases decreased from 244 million to 216 million; moreover, malaria mortality rates showed a global reduction of 26% between 2000 and 2010.

Malaria in humans is caused by 5 *Plasmodium* parasites: *Plasmodium falciparum*, *P. vivax*, *P. malariae, P. ovale* and *P. knowlesi*. The current distribution of human-pathogenic *Plasmodium* species shows preponderance of *P. falciparum* in tropical Africa, while *P. vivax* prevails over *P. falciparum* in South America. Both *P. falciparum* and *P. vivax* are prevalent in south-eastern Asia and western Pacific. Although *P. malariae* may occur in all malarious areas, its prevalence is generally low. In tropical Africa, *P. falciparum* and *P. malariae* co-infection is sometimes encountered. *P. ovale* is widespread principally in tropical Africa whereas *P. knowlesi* infection occurs only in certain forested areas of South-East Asia.

Malaria burden is hard to estimate, particularly in low income countries where data collection and reporting quality is poor. Incomplete and discontinuous reports from single health facilities may alter final global malaria prevalence. Malaria cases are often under-diagnosed in hyper endemic countries, where mild symptoms of chronic malaria my possibly lead to misdiagnosis. On the contrary, over-diagnosis may also occur. In fact, not all reported malaria cases are confirmed by microscopy or others assay, such as rapid diagnostic tests (RDTs). Furthermore, in hyper endemic areas febrile illnesses from different causes might be misdiagnosed with malaria.^3^ Anyway, WHO guidelines recommends that microscopy or RDTs should be used to confirm all malaria cases.

Another issue is the lack of population denominator that makes the real incidence of malaria difficult to assess. Data emerging from WHO reports just estimate malaria incidence and mortality, reporting malarial cases and malarial death from the different WHO regions, collected by Ministries of Health of different countries. These data do not reflect the real incidence in the general population. Nevertheless, they are good indicators to assess malarial control programmes and to estimate the impact of malaria infection in health systems.

## Malariometry

The term *endemicity* is a proxy to indicate disease prevalence. Areas presenting the same level of endemicity often have similar characteristics of disease distribution, guiding malaria experts to design, implement and monitor control and prevention activities.^4^

Malaria endemicity is a very complex issue, that is influenced by factors related to the man-host interactions (agricultural activities, nocturnal activities, migration movements, wars, limited resources), to the parasite (different species, sporogonic cycle length, drug susceptibility), to the vector (density, larvae breeding sites, temperature, receptivity, feeding pattern, longevity, insecticide susceptibility) and to the environment (physical – biological – socio-economic). The detailed analysis of all such variables is beyond the scope of this brief article that will only report available data, referring the interested reader to more exhaustive and valuable treatises on the topic.

**Figure 2:**
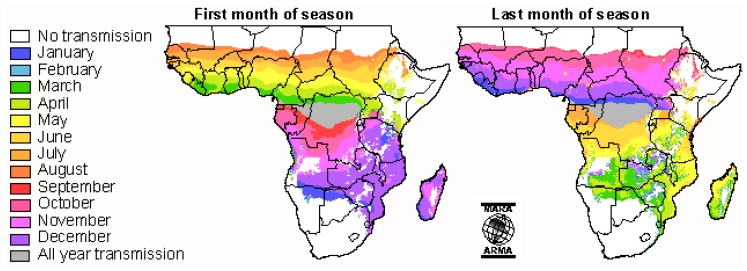
Illustration show the start/end of the transmission season in Africa (www.mara.org.za)

Moreover, malaria incidence may fluctuate according to seasonality. Figure 2 shows the months of start and end of malaria transmission over the year in the African continent.

Different methods to classify malaria endemicity in a population exist. These methods includes (i) proportion of individuals in a population with a palpable enlargement of spleen (*spleen rate* [SR]), (ii) proportion of individuals in a population with a laboratory-confirmed parasite infection (*parasite rate* [PR]), (iii) number of infective bites per person (*entomological inoculation rate* [EIR]) and (iv) number of microscopically confirmed malaria cases detected during one year per unit population (*annual parasite incidence* [API]).^5^

Proportion of individuals with splenomegaly (SR) in a given population was the first method used to assess malaria endemicity during a malariometric survey in 1848 in India, where spleen dimension was assessed in selected population age groups. Thus, malariometry attention was focused on clinical manifestations of malaria. On the basis of splenomegaly prevalence rates in children from 2 to 9 years old, 4 different endemicity areas can be distinguished: *holo-endemic areas*, where proportion of people with splenomegaly is above 75%; *hyper-endemic areas*, where splenomegaly prevalence is between 51 and 75%; *meso-endemic areas*, with prevalence between 50 and 11%; *hypo-endemic areas*, where prevalence is below 11%.^5^

Parasite rate (PR) assesses the proportion of individuals with microscopically confirmed presence of asexual parasites in peripheral blood. It’s a technique that requires expert laboratory technicians and suffers of malaria seasonal variation.

Spleen and parasite rate are actually less used, whereas entomological inoculation rate (EIR) and annual parasite incidence (API) are utilized to prepare epidemiologic malaria maps that show malaria distribution in the world. Where data are unavailable, a model is required to predict malaria endemicity.^6^–^10^ Many recent studies investigated a predictive framework known as model-based geostatistics (MBG) to asses malaria endemicity^11^–^15^ and the prevalence of other vector-borne and intermediate host borne diseases.

Maps showing the global distribution of *P. falciparum* and *P. vivax* has recently been published by Malaria Atlas Project. These maps provide a geographical framework for monitoring malaria incidence and evaluation of impact on malaria control worldwide (Figures 3a and 3b).

*P. falciparum* malaria endemicity has been mapped considering national malaria reports, medical intelligence and biological rules of transmission, such as temperature and aridity, important for *Anopheles* vectors spreading^16^,^17^. Thus, in 2007, the world was stratified into three spatial representation: (i) areas without *P. falciparum* malaria risk, (ii) unstable risk areas (*P. falciparum* annual parasite incidence [PfAPI]: < 0.1 per 1.000 people per annum [pa]) and (iii) stable risk areas (PfAPI ≥ 0.1 per 1.000 people pa).^18^ The global area at risk of stable *P. falciparum* malaria was quantified in 29.7 million km^2^, distributed into Africa (18.2 million km^2^, 61.1%), Americas (6.0 million km^2^, 20.3%) and Central and South East Asia regions (5.5 million km^2^, 18.6%). Of the 2.37 billion people exposed to *P. falciparum* transmission worldwide, 0.98 billion live in unstable risk areas,^16^, ^17^ whereas 1.383 billion live in stable risk areas, distributed into Africa (0.657 billion, 47.5%), Americas (0.041 billion, 2.9%) and Central and South East Asia (0.686 billion, 49.6%). Children are the most represented category, accounting for 32% of the population at risk in Americas and in Central and South East Asia. In Africa this percentage rise up to 43%.

*P. vivax* is transmitted in 95 tropical, subtropical and temperate countries.^19^ People living at risk of *P. vivax* malaria infection are 2.85 billion, 91% living in Central and South East Asia region, 5.5% in America and 3,4% in Africa. As many as 57.1% of people exposed to *P. vivax* infection lives in unstable malaria areas.

*Stable - unstable* classification is another way to determine malaria endemicity. Macdonald defined malaria stability on the ground of the number of mosquitoes lifetime bites in the human host.^20^ This vector-based index differentiated stable and unstable malaria. Vector-based classification is less used because of entomological-based metrics complexity, ethical concerns related to exposing human beings to malaria infection and measurement error issues.^5^

## Endemic Areas Distribution

Different malaria endemic areas have different epidemiological situations and also the feasible targets may differ. According to WHO, the following terminology should be adopted when referring to malaria endemic status: *Malaria control*: reducing the malaria disease burden to a level at which it is no longer a public health problem.

*Malaria elimination*: the interruption of local mosquito-borne malaria transmission; reduction to zero of the incidence of infection caused by human malaria parasites in a defined geographical area as a result of deliberate efforts; continued measures to prevent reestablishment of transmission are required.

*Malaria eradication*: permanent reduction to zero of the worldwide incidence of infection caused by a particular malaria parasite species. Intervention measures are no longer needed once eradication has been achieved.

Hereunder we summarize data from WHO Reports, showing malaria trends in different WHO Regions. Trends in malarial cases and deaths reflect control programmes, such as distribution of insecticide-treated nets (ITN), long lasting insecticidal nets (LLIN), use of indoor residual spraying (IRS) and artemisinin combination treatment (ACT).

On the ground of slide positivity rate (SPR) and of the population at risk of malaria, WHO distinguishes areas with advance malaria control activities in (I) *pre-elimination phase*, (II) *elimination phase*, (III) *prevention of reintroduction* and (IV) *malaria-free stages* as shown in table 2.^1^

Most malaria cases and deaths occur in the *African Region*. As a consequence of implementation programs, high burden countries of African Region, such as Madagascar, Sao Tome and Principe, Eritrea, Rwanda and Zambia, showed a decrease in malaria cases up to 50% between 2000 and 2009.^21^ Rwanda showed a decrease by 74% of confirmed malarial cases between 2005 and 2010 and slide positivity rate decreased from 35% to 9%. Moreover, number of malaria hospital admissions and malaria deaths showed a decrease of 65% and 55% respectively. Zanzibar, belonging to United Republic of Tanzania, showed a dramatic decrease of malaria admissions and deaths due not only to the efficacy of control strategies, but also to favourable geographic position. In low-transmission countries of African Region control strategies have also been performed. Thanks to these strategies, Algeria is in the malaria elimination phase, Capo Verde in pre-elimination phase.^1^

In 15 countries of the WHO *Region of the Americas*, where *P. vivax* is the most represented species, reductions of more than 50% in the number of the reported cases were observed. During 2010, malaria transmission occurred in 21 countries, of which 17 are in the control stage and 4 are in the pre-elimination stage. Bahamas and Jamaica are in the prevention of reintroduction phase. In Ecuador, malaria cases dropped from 105.000 in 2000 to 4.120 in 2009, a reduction of 96% due to IRS, LLINs distribution, strengthening of malaria diagnosis and treatment and also due to Global Found, UNICEF, USAID and government funds invested in malaria control.^1^

In 2010, 2.4 million confirmed malaria cases were reported in WHO *South-East Asia Region*. India accounts for 66% of confirmed cases, even though a reduction of 28% of the cases between 2000 and 2010 was observed. In 2010, malaria deaths were 2.426 as reported from eight countries of the region, most of all reported in India. Democratic People’s Republic of Korea and Sri Lanka are actually in pre-elimination phase. Bangladesh, Bhutan, the Democratic Republic of Timor-Leste, India, Indonesia, Myanmar, Nepal and Thailand are in the control phase.

In the WHO *European Region*, the number of autochthonous cases decreased from 32.394 in 2000 to 176 in 2010. All malaria cases are now attributable to *P. vivax* infection; no *P. falciparum* cases occurred since 2008. Malaria cases were identified in Azerbaijan, Kyrgyzstan, Tajikistan, Turkey and Uzbekistan. Georgia reported no cases in 2010 and Turkmenistan was declared malaria-free in October 2010. A particular case is represented by Greece, a country that was declared malaria-free from 1974.

Since June 2011 a total of 63 autochthonous malaria cases have been reported,^21^ all due to *P. vivax* infection. Cases occurred mostly in the southern region of the country, specifically of the Evrotas delta area of Laconia district in agricultural area with large migrant populations.^21^,^22^ Other cases occurred in the Evia/Euboea (island east of the Central Greece region), Eastern Attiki, Voitia and Larissa districts.^21^

In the WHO *Eastern Mediterranean Region*, Islamic Republic of Iran and Saudi Arabia are in the elimination phase, while Egypt, Iraq, Oman and Syrian Arab Republic are in prevention of reintroduction phase. Morocco was confirmed malaria-free in May 2010. Afghanistan, Djibouti, Pakistan, Somalia, Sudan, South Sudan and Yemen are in the control stage, and they still represent high malaria transmission areas.

As many as 262.000 confirmed cases were reported from the WHO *Western Pacific Region* in 2010. Papua New Guinea, Cambodia and Solomon Island account for 70% of these malarial cases. China, Philippines, Republic of Korea and Vietnam showed a decrease in malaria cases up to 50% between 2000 and 2010, while other countries showed a more slowly decrease (e.g. Cambodia, Lao People’s Democratic Republic, Malaysia, Solomon Island, Vanuatu).

## Plasmodium Species Distribution

*Plasmodium* species are differently distributed in the world. Prevalence of malaria cases and deaths differs during different seasons, as mentioned before. Prevalence data must be related to season and to endemicity of each countries.

The following paragraphs report available epidemiological data obtained in different geographical areas for the different Plasmodia species. While recognizing the value of the robust epidemiological research reviewed, we would like to underline that comparison among studies is virtually impossible because of lack of homogeneity in diagnostic methods, seasonality and methodological approach. The interested reader is then invited to refer to the selected articles for further information and details.

- *Plasmodium falciparum*
  *P. falciparum* is widespread in nearly all malaria endemic countries. A study identified 2.37 billion people at risk of *P. falciparum* transmission worldwide, 26% located in the African Region and 62% in South Eeast Asian and Western Pacific regions.^17^ In Africa, many epidemiological studies suggest that *P. falciparum* is the most prevalent malarial species. Blood samples were collected between 1998 and 2006 from nine different African countries and analyzed by PCR for the presence of each of the four human malaria parasites.^23^ Out of 2.588 samples, 1.737 were positive for *Plasmodium* species and 1.711 (98,5%) were positive for *P. falciparum* considering both mono and mixed infection. Another study performed in 4 villages in Mulanda sub-county, in eastern Uganda, showed a prevalence of *P. falciparum* infection of 94% during rainy season, from July to December, using thin film diagnosis.^24^ A study performed in metropolitan Lagos, Nigeria, showed a microscopic prevalence of *P. falciparum* species of 88,5% in pregnant women attending antenatal care clinic, during one observation year.^25^ In Asia, *P. falciparum* and *P. vivax* are the two prevalent species. In India, *P. falciparum* is mostly widespread in Orissa state^27^,^28^ while in the west of the country mixed infections are predominant.^27^ In Bangladesh, samples collected during 3 years from febrile patients and analyzed by species-specific PCR showed a *P. falciparum* prevalence of 81,5%.^29^ In Cambodia, *falciparum* prevalence among residents of 8 villages was about 59% using a new PCR technique.^30^ In Thailand (Tak, Chantaburi, Prachuab Khirikhan, Yala and Narathiwat Provinces), PCR research of *P. falciparum* among febrile patients from October 2006 to September 2007 showed a prevalence of 43,5% both in mono and in mixed infection.^31^ Samples collected from 146 selected patients with uncomplicated malaria in 2008 in southern Myanmar underwent PCR analysis to investigate malaria parasites: the prevalence of *P. falciparum* was 52,1% considering mono and mixed infection.^32^ In South America *P. vivax* is the predominant species, followed by *P. falciparum* (25.7%).^33^ Most of the malaria cases occur in Brazil; the others are distributed in 20 other countries of Central and South America.^33^
- *Plasmodium vivax*
  In Central and Western Africa *P. vivax* infection is rare because of the high prevalence of the red blood cells Duffy negative phenotype in the population, interfering with *P. vivax* merozoite entry into the red blood cells. A large study carried out in nine African countries failed to detect *P. vivax* species.^23^ Despite these studies, however, there are some evidence of *P. vivax* transmission in West and Central Africa. In Congo, specific *P. vivax* antibodies were researched in 409 samples from patients coming from an health center located on the west coast, where Duffy antigen is expected to be > 95%. Out of the 409 samples, 55 (13%) tested positive for specific *P. vivax* antibodies.^34^ Another study from Kenya demonstrated the presence of *P. vivax* among mosquitoes; in addition, *P. vivax* DNA was amplified and sequenced in blood of two Duffy negative children.^35^ In eastern and southern Africa only 5% of malaria infections are attributable to *P. vivax*.^36^ In Asia, *P. vivax* and *P. falciparum* are the predominant species.^37^ In India, isolate *P. vivax* infection is widespread in the southern state of Tamil Nadu, while mixed-species infections are prevalent in the west.^27^ In Bangladesh, samples collected from febrile patients underwent PCR research of *Plasmodium* species, showing a prevalence of *P. vivax* infection of 15,3% in mono-infected patients and of 27,5% in mixed infections.^29^ In Cambodia, prevalence of *P. vivax* infection detected by PCR method in samples collected in September 2001 was 15%.^30^ Studies performed in Thailand and in Myanmar showed that *P. vivax* is the most prevalent malarial species,^31^,^32^ such as in the WHO Eastern Mediterranean Region, in particular in Afghanistan, Islamic Republic of Iran and Turkey.^38^–^41^ In Central and South America *P. vivax* is the predominant *plasmodium* species, accounting for 71–81% of all malaria cases.^33^,^35^ Studies demonstrate that *P. vivax* accounts for 83,7% of malarial infections in Brazil,^43^ for 70% of infections in Colombia^44^ and for 90% of infections in Ecuador.^45^
- *Plasmodium ovale*
  The real burden of *P. ovale* malaria is difficult to asses because its diagnosis is difficult. *Plasmodium ovale* may be encountered in sub-Saharan Africa and in Asia.^46^ A recent study from Mozambique tested malaria prevalence among febrile patients: only 2 of 111 malaria positive patients presented *P. ovale* mono-infection, while 4 *P. ovale* and *P. falciparum* mixed infections were also detected.^47^ In Congo, a cross-sectional, population-based cluster household survey of adults aged 15–59 years demonstrated that *P. ovale* parasitemia was rare and its prevalence in mono-infection was only 0,1%.^26^ In a recent multicenter study, blood samples were collected from the indigenous population of nine African countries and malaria parasites were searched by PCR method. Of 1.737 samples, 67 were positive for *P. ovale:* 12 single infections, 51 mixed with *P. falciparum* and 4 triple infections with *P. falciparum* and *P. malariae*. When samples from Rwanda, Mozambique, Angola and Sao Tome were excluded, *P. ovale* infection represented 3,9% of all malaria cases.^23^ Another study performed in Congo-Brazzaville, Uganda and Equatorial Guinea, concluded that two *P. ovale* species, *P. o. curtisi* and *P. o. wallikeri*, are both widespread in Africa; in Uganda and Equatorial Guinea the prevalence of *P. ovale* spp. in population-based samples was found to be between 1% and 6%.^48^ There are many evidence of *P. ovale* infection in Asia. Samples collected during 2007/2008 in Bangladesh from 379 febrile patients who underwent microscopic, DNA extraction and nested PCR analysis: 3 of the 189 positive samples (1,6%) were positive for *P. ovale*.^29^ *Nested* PCR detected *P. ovale* parasites in 1,3% of blood samples collected from 1.356 inhabitants of eight villages of Rattanakiri Province (Cambodia).^30^ In a study performed in Myanmar, *P. ovale* was detected by PCR technique in 4,9% of malaria positive samples; most of cases were co-infections with *P. falciparum*, *P. vivax* and/or *P. malariae*.^49^ Case reports of *P. ovale* infection were recently published from Gujarat, India,^50^ Malaysia^51^ and Sri Lanka.^52^ *P. ovale* infection is present in Papua, Indonesia^53^ and in Thailand^31^, while it is very rare in Philippines, where has been reported only in the island of Palawan.^54^
- *Plasmodium malariae*
  *P. malariae* is spread in sub-Saharan Africa, in southeast Asia, in Indonesia, in many islands in western Pacific and in areas of the Amazon Basin of South America. Its distribution overlaps with that of *P. falciparum*.^55^ In a recent study, blood samples were collected from the indigenous population of nine African countries and malaria parasites were searched by PCR method. *Plasmodium malariae* was found in 147 of the 1.737 positive blood samples, 14 as mono-infections, 129 as mixed infections with *P. falciparum* and 4 as triple infections with *P. ovale* and *P. falciparum*.^23^ Excluding samples from Rwanda, Mozambique, Angola and Sao Tome, *P. malariae* infections represented 8.5% of all malaria infections.^23^ In Nigeria, between November 2001 and October 2002, a total of 350 pregnant women attending the antenatal clinics were randomly recruited and blood samples were collected. Of 350 blood samples, 96 (27,4%) were positive for malaria parasite and 11 (11,5%) were *P. malariae* positive as tested by microscopy.^25^ During the rainy season, blood samples were collected from resident people of four village in Mulanda, Uganda. Of 709 malaria positive samples, 6% were positive for *P. malariae*.^24^ In the state of Orissa, India, the prevalence of *P. malariae* detected by PCR method was 44,6% during the peak season of malaria incidence.^28^ A recent case report was published demonstrating the presence of *P. malariae* in Bangladesh.^56^ In Papua New Guinea, prevalence of *P. malariae* was detected by *nested* PCR, by quantitative PCR and by PCR-ligase detection reaction-fluorescent microsphere assay (PCR-LDR-FMA); the results were 3,3%, 4,7% and 7,7% respectively.^57^ *Plasmodium malariae* infection was confirmed in Thailand^58^ and in Yemen.^42^ *P. malariae* is present in Brazil, in French Guiana and in Venezuela.^43^,^59^,^60^ A report recently published confirmed *P. malariae* presence in Haitian refugees in Jamaica.^61^
- *P. knowlesi*
  *Plasmodium knowlesi* infection is localized only in the South East Asia Region and interest both monkeys, where it was first reported, and humans. Forest areas are the *reservoirs* of *P. knowlesi*, that was first reported in humans in 1965 in a man who had worked in the jungle of Pahang, Peninsular Malaysia. An analysis of stored blood films identified cases of *Plasmodium knowlesi* infection occurring since 1996 in Sarawak region, Malaysian Borneo.^62^ In Sabah region, Malaysian Borneo, samples were collected from February to November 2010 from patients with suspected malaria. *Nested* PCR was performed in all 243 samples collected; of 107 samples positive for malaria parasite, 63 were positive for *P. knowlesi*, demonstrating an high incidence of *P. knowlesi* infection in the interior division of Sabah.^63^ Another study performed in Thailand screened 1.874 samples collected from febrile patients during a period of one year. Of the 1.751 sample positive for malaria parasites, 10 were positive for *P. knowlesi,* that mostly occurred in males with uncomplicated malaria coming from southern and southwestern regions of Thailand.^31^ *P. knowlesi* mono and mixed infection were also demonstrated in Myanmar,^32^ in the Philippines^64^ and in Singapore.^65^ According to a three years prospective study performed in Cambodia, two *P. knowlesi* malaria cases were diagnosed by *nested* PCR.^66^ A recent study performed in Vietnam showed the presence of *P. knowlesi* in three of ninety-five samples of patients with *P. malaria* mono or mixed infection, demonstrating for the first time the presence of *P. knowlesi* malaria in Vietnam.^67^

## *Anopheles* Vectors

Malaria is transmitted exclusively through the bites of *Anopheles* mosquitoes. There are 512 *Anopheles* species recognized worldwide and 50 only provisionally designated and awaiting description.^68^ Seventy *Anopheles* species are able to transmit *Plasmodium* parasite to human hosts.^69^ *Anopheles* mosquitoes breed in water and each species has its own breeding preference. Transmission is more intense in places where mosquito lifespan is longer (parasite has time to complete its development inside the mosquito) and where anthropophilic mosquitoes prevail. Forty-one of the 512 *Anopheles* species are defined by experts “Dominant Vector Species” (DVS). DVS are the most important malarial vector worldwide, providing the majority of human malaria cases. Characteristics of dominant vector species are their propensity for humans feeding, longevity, abundance and elevate vectorial capacity.^70^ Many studies were recently conducted to define *Anopheles* distribution in Africa,^71^,^72^ Americas,^73^,^74^ Europe,^75^ Central and South East Asia^76^ and worldwide.^77^

Africa has the most effective and efficient DVS of human malaria, the *Anopheles gambiae complex*; thus some areas account the highest entomological inoculation rates and the highest malaria prevalence worldwide.^17^,^78^,^79^ There are 4 principal species belonging to *An. gambiae complex: An. gambiae, An. arabiensis, An. merus* and *An. melas*. Other three highly anthropophilic DVS are spread in African region: *An. funestus, An. moucheti* and *An. nili*.^80^

Despite European and the Middle Eastern regions are low malaria transmission areas, existence of DVS may play a potential role in re-introduction of malaria.^78^,^81^ *An. atroparvus* is mostly diffused in these regions.^80^ Nine DVS were found in the Americas, where *An. darlingi* is considered one of the most efficient malaria vectors.^82^

In Asian-Pacific region 19 DVS were found; some of them are predominant in the Arabian Peninsula (e.g*. An. stephensi* and the *An. culicifacies complex*), others in the Indian subcontinent, China and Korea (e.g. *An. lesteri*), in the Solomon Islands and Vanuatu (e.g. the *An. farauti complex*) and finally in Queensland and the Northern Territory of Australia (e.g. the *An. farauti complex*). Myanmar appeared to contain the greatest number of DVS, of wich *An. aconitus*, *An. annularis*, *An. barbirostris complex*, *An. culicifacies complex*, *An. dirus complex*, *An. maculatus group*, *An. minimus complex*, *An. sinensis complex*, *An. stephensi*, *An. subpictus complex* and, in some coastal site, *An. sundaicus complex*.^83^

Environmental factors play an important role in vector distribution and malaria biodiversity. Climate seasonality, rainfall patterns, temperature, humidity, presence of vegetation and surface water all are directly related to the malaria transmission cycle. In addition, human activities such as agriculture, irrigation, deforestation, urbanization, population movements, dam/road constructions and wars are also connected to transmission levels and malaria epidemiology.^84^

## Conclusion

Malaria is still considered a global health problem and a major killer. Morbidity and mortality burden of malaria could be reduced strengthening prevention, improving malaria diagnosis, using correct therapies based on artemisinin combination and adopting strategies aimed at preventing drug resistances.

Real malaria incidence is difficult to obtain. However, it is possible to make reliable estimates thanks to the data supplied by Ministries of Health of different countries and to accurate prevalence studies. Determination of real incidence of malaria and determination of the real *Plasmodium* species distribution are two different issues that could help expert in eradication of malaria, the real and unique goal in the fight against malaria.

## Figures and Tables

**Figure 3a:**
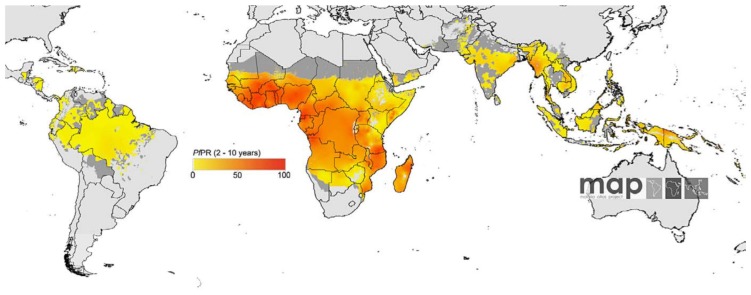
*P. falciparum* distribution (www.map.ox.ac.uk).

**Figure 3b:**
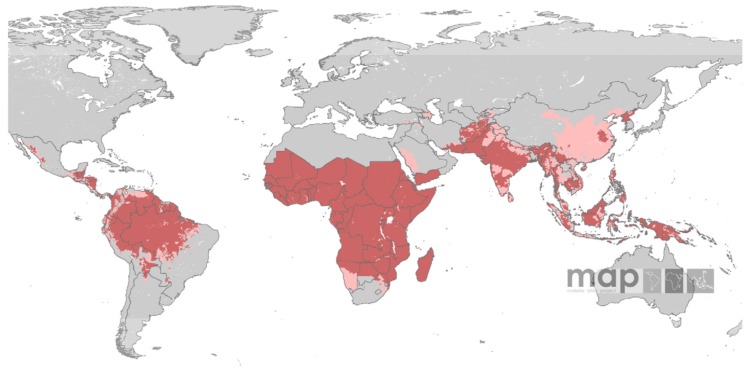
*P. vivax* distribution (www.map.ox.ac.uk).

**Table 1:** Malaria cases and death by geographical region (World Malaria Report 2011, modified)

WHO region	Malaria cases (%)	Malaria deaths (%)
African Region	81	91
South East Asia	13	6
Eastern Mediterranean Region	5	3
Others	1	<1

**Table 2:** classification of selected countries in the pre-elimination, elimination, prevention of reintroduction and malaria-free stages, as 1 December 2011 (Ref. [1]).

WHO Region	Pre-elimination	Elimination	Prevention of reintroduction	Certified malaria-free within last 5 years, or no local transmission reported for over a decade
Africa	Cape Verde	Algeria		
Americas	Argentina, El Salvador, Mexico, Paraguay		Bahamas, Jamaica	
Eastern Mediterranean		Iran, Saudi Arabia	Egypt, Iraq, Oman, Syrian Arab Republic	Morocco, Turkmenistan, United Arab Emirates
Europe		Azerbaijan, Kyrgyzstan, Tajikistan, Turkey, Uzbekistan	Georgia, Russian Federation	Armenia
South East Asia	DPR Korea, Sri Lanka			
Western Pacific	Malaysia	Republic of Korea		
